# Developing a Core Outcome Set for the Evaluation of Antibiotic Use in Prelabor Rupture of Membranes: A Systematic Review and Semi-Structured Interview

**DOI:** 10.3389/fphar.2022.915698

**Published:** 2022-08-01

**Authors:** Dan Liu, Lin Wu, Jiefeng Luo, Siyu Li, Yan Liu, Chuan Zhang, Linan Zeng, Qin Yu, Lingli Zhang

**Affiliations:** ^1^ West China School of Pharmacy, Sichuan University, Chengdu, China; ^2^ Department of Pharmacy, West China Second University Hospital, Sichuan University, Chengdu, China; ^3^ Evidence-Based Pharmacy Center, West China Second University Hospital, Sichuan University, Chengdu, China; ^4^ Key Laboratory of Birth Defects and Related Diseases of Women and Children, Sichuan University, Ministry of Education, Chengdu, China; ^5^ Department of Obstetrics, West China Second University Hospital, Sichuan University, Chengdu, China; ^6^ West China School of Medicine, Sichuan University, Chengdu, China; ^7^ National Drug Clinical Trial Institute, West China Second University Hospital, Sichuan University, Chengdu, China

**Keywords:** core outcome sets, outcome reporting, pregnancy, prelabor rupture of membranes, systematic review, semi-structured interview

## Abstract

**Background:** Prelabor rupture of membranes (PROM) is associated with maternal and neonatal infections. Although guidelines suggest prophylactic antibiotics for pregnant women with PROM, the optimal antibiotic regimen remains controversial. Synthesizing the data from different studies is challenging due to variations in reported outcomes.

**Objective:** This study aimed to form the initial list of outcomes for the core outcome set (COS) that evaluates antibiotic use in PROM by identifying all existing outcomes and patients’ views.

**Methods:** Relevant studies were identified by searching PubMed, EMBASE, Cochrane Library, Chinese National Knowledge Infrastructure, Wanfang, and VIP databases. We also screened the references of the included studies as a supplementary search. We extracted basic information from the articles and the outcomes. Two reviewers independently selected the studies, extracted the data, extracted the outcomes, and grouped them into domains. Then, semi-structured interviews based on the potential factors collected by the systematic review were conducted at West China Second Hospital of Sichuan University. Pregnant women who met the diagnostic criteria for PROM were enrolled. Participants reported their concerns about the outcomes. Two researchers identified the pregnant women’s concerns.

**Results:** A total of 90 studies were enrolled in this systematic review. The median outcomes in the included studies was 7 (1–31), and 109 different unique outcomes were identified. Pre-term PROM (PPROM) had 97 outcomes, and term PROM (TPROM) had 70 outcomes. The classification and order of the core outcome domains of PPROM and TPROM were consistent. The physiological domain was the most common for PPROM and TPROM outcomes. Furthermore, 35.1 and 57.1% outcomes were only reported once in PPROM and TPROM studies, respectively. Thirty pregnant women participated in the semi-structured interviews; 10 outcomes were extracted after normalized, and the outcomes were reported in the systematic review. However, studies rarely reported pregnant women’s concerns.

**Conclusion:** There was considerable inconsistency in outcomes selection and reporting in studies about antibiotics in PROM. An initial core outcomes set for antibiotics in PROM was formed.

## 1 Introduction

Prelabor rupture of membranes (PROM) is a rupture of membranes before the onset of labor, which consists of “pre-term prelabor rupture of membranes (PPROM)” and “term prelabor rupture of membranes (TPROM)” (Siegler et al., 2020). It affects 2.3%–18.7% of pregnancies and increases the risk of intrauterine infection, neonatal sepsis, neonatal pneumonia, etc. (Kenyon et al., 2001a; Martin et al., 2005; Mercer, 2005; Smith et al., 2005; Clark and Varner, 2011; Reuter et al., 2014; Middleton et al., 2017; Zhuang et al., 2020). Although guidelines suggest that the use of prophylactic antibiotics could reduce infection morbidity and improve the outcomes for mothers and newborns, the optimal antibiotic regimen is still controversial (Yudin et al., 2009; Kenyon et al., 2013; Thomson and Royal College of Obstetricians and Gynaecologists, 2019; Chatzakis et al., 2020; Siegler et al., 2020). Despite many studies about the antibiotics regimens for PROM conducted, it is difficult to synthesize their data due to outcome variations. As a recent systematic review shows, only 70.0% (17/20) of the included studies reported the primary outcome. The risk of bias was 35.0% (7/20) and 90.0% (18/20) of the included studies, including risk in “Measurement of outcome” and “Selection of reported result,” respectively (Chatzakis et al., 2020).

A core outcome set (COS), defined as an agreed standardized set of outcomes that should be measured and reported as a minimum, could improve consistency in outcome measurement and reduce outcome reporting bias. A COS would eliminate unnecessary waste in producing and reporting research findings (Williamson et al., 2012). The COS is drawing increasing attention across all health research areas and is referred to as a starting point for outcome selection in the work of some trialists, systematic reviewers, and guideline developers (COS users) (Gorst et al., 2016).

However, there is no COS for antibiotics in PROM or COS for treating or preventing infection in pregnant women. This systematic review and semi-structured interview would form the initial list of outcomes for the COS of antibiotics in PROM by identifying all existing outcomes and patients’ views.

## 2 Methods

This COS project is registered on the core outcome measures in effectiveness trials (COMET) database, and further details are available at https://www.comet-initiative.org/Studies/Details/1986.

### 2.1 Systematic Review

The part of the systematic review was performed and reported per the Preferred Reporting Items for Systematic Reviews and Meta‐Analyses guidelines for systematic reviews (Preferred Reporting Items for Systematic Reviews and Meta-Analyses, 2009).

#### 2.1.1 Search Strategy

We conducted an electronic search of PubMed, EMBASE, Cochrane Library, Chinese National Knowledge Infrastructure, Wanfang, and VIP Database from inception to September 2021. The search strategy was adjusted specifically for each database. It combined medical subject headings and free text terms for (“Fetal Membranes, Premature Rupture” “antibiotics” or “Prelabor rupture of membranes”) and (“Anti-Infective Agents” or “antibiotics” or “Penicillins” or” Cephalosporins” or” azithromycin” or” erythromycin” or” Clindamycin” ). Supplementary Table S1 lists the search terms. Citation lists of the included studies were reviewed to identify any intervention reports missed by the search strategy.

#### 2.1.2 Inclusion Criteria

The following studies were included: 1) Participants: pregnant women (no restriction for gestational age) met the diagnostic criteria for PROM according to the guidelines of the Chinese Medical Association, American College of Obstetricians and Gynecologists, Society of Obstetricians & Gynaecologists (SOGC), Royal College of Obstetricians and Gynaecologists (ROGC), etc. 2) Intervention: antibiotics. 3) Type of study: systematic reviews, randomized controlled trials, non-randomized controlled trials, or cohort studies. The following studies were excluded: 1) non-Chinese and non-English literature, 2) unobtainable full-texts.

#### 2.1.3 Data Extraction

Titles and abstracts were independently screened by two reviewers to determine potential eligible studies, and full texts of potentially relevant articles were independently screened by two reviewers to assess for eligibility. Disagreements were resolved by consensus or consulted a third reviewer. Two reviewers independently extracted data from the included studies and cross-checked it. The extracted data included: 1) the basic information of the articles (the first author, published year, study design, country, etc.); 2) the characteristics of participants and interventions; 3) the outcomes reported (names, definitions, and measurements of each outcome).

#### 2.1.4 Assessment of Risk of Bias

There was no assessment of the risk of bias since the purpose of this study was to identify all outcomes reported irrespective of the study quality.

#### 2.1.5 Data Synthesis

All outcomes were extracted verbatim from studies. Variations in the same outcome reporting were revised for consistency, and the composite outcomes were split into unique outcomes by a researcher with clinical experience in obstetrics. Outcome terminologies were assigned to one of the core outcome domains according to the COMET Handbook (Williamson et al., 2017). We calculated the number of unique outcomes for each study and outcome domain, the number of reported studies for each outcome, and the median number of the reported studies for each outcome domain.

### 2.2 Semi-Structured Interview

According to recommendations of COS-STAndards for Development and COMET handbook (version 1.0) (Kirkham et al., 2016; Williamson et al., 2017), a list of outcomes from published clinical trials may be supplemented with semi-structured interviews with patients. Therefore, we conducted the semi-structured interview to obtain the opinions of patients on PROM treatment.

The semi-structured interview study was conducted at West China Second Hospital of Sichuan University from January to February 2022. The West China Second University Hospital, Sichuan University, provided ethical approval. The participants gave verbal consent before their interviews. The participants’ socioeconomic information of participants came from the hospital information system.

#### 2.2.1 Participants

Pregnant women in West China Second Hospital of Sichuan University, January to February 2022, who met the diagnostic criteria for PROM were enrolled. The exclusion criteria included: 1) pregnant women with serious illnesses who were not suitable to participate in the study; 2) pregnant women with communication difficulties; 3) pregnant women who refused to participate. The sample size was 30 since 30 subjects could achieve data saturation reported in other studies (Keyvanara et al., 2013; Alkadhimi et al., 2020). However, if new information is generated in the final interview, the sample size of the interview will increase.

#### 2.2.2 Procedure

The research team designed a semi-structured interview guide involving open-ended questions (Supplementary). The face-to-face semi-structured interviews took place at the patient’s bedside at mutually convenient times. The researchers would explain the content and purpose of the study to the patients and interview them after obtaining their informed consent. Interviews were digitally audio-recorded using a mobile phone.

#### 2.2.3 Analysis

All the interviews were transcribed literally by a researcher. Our systematic review developed a consensus codebook using a deduction coding process and evaluating the first 10 transcripts to identify emerging codes through an inductive coding process. Each transcript was independently coded by two researchers, and coding inconsistencies were resolved by discussion. Disagreements were resolved by consensus or a discussion in the research group. Data analysis was processed by identifying the codes to judge whether these were new outcomes and whether they should be added to the list of candidate outcomes. We would identify whether these outcomes are new and judge whether they should be added to the list of candidate outcomes.

## 3 Results

### 3.1 Systematic Review

#### 3.1.1 Study Characteristics

**FIGURE 1:**
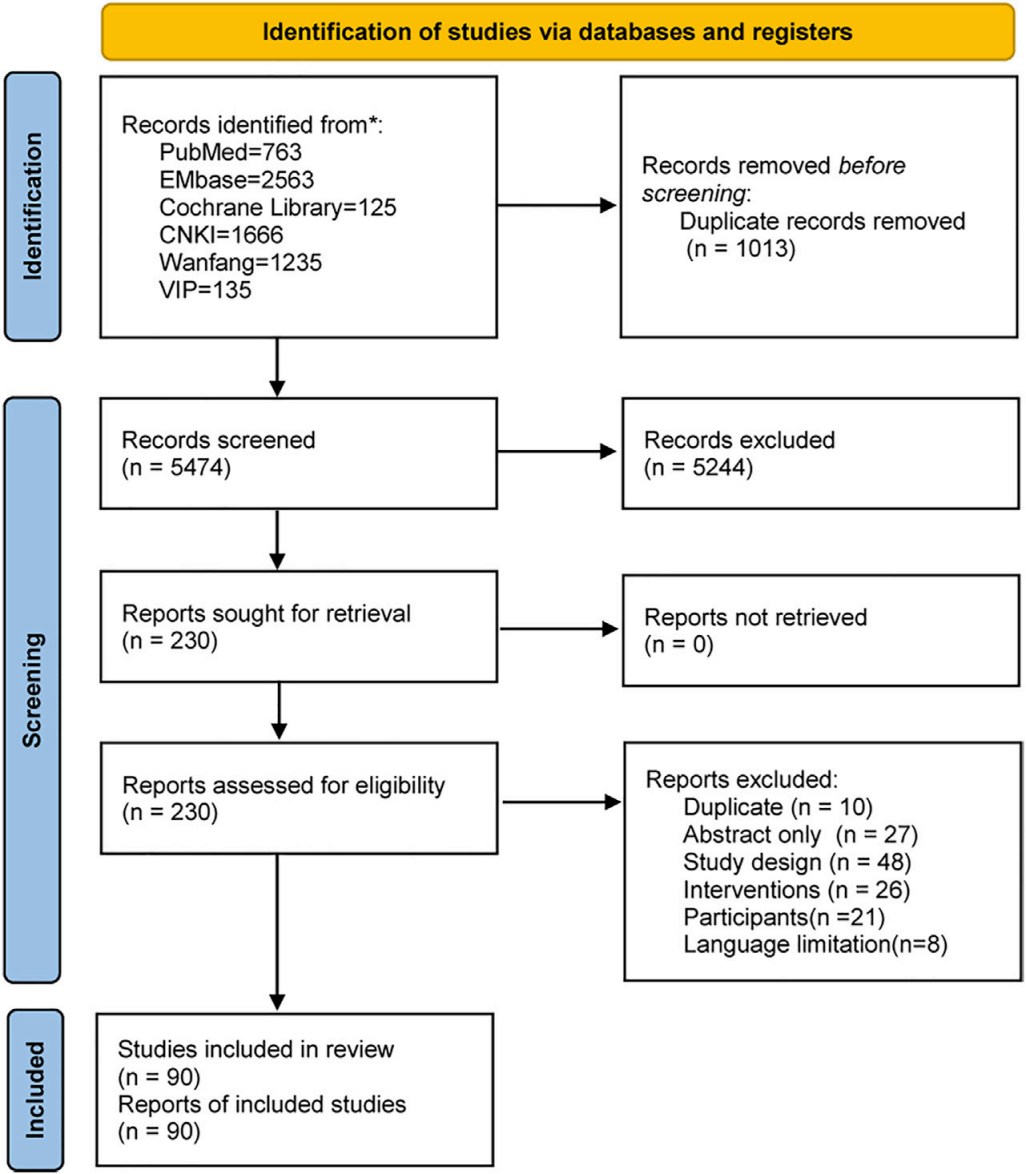
Flow diagram of study selection.

**FIGURE 2:**
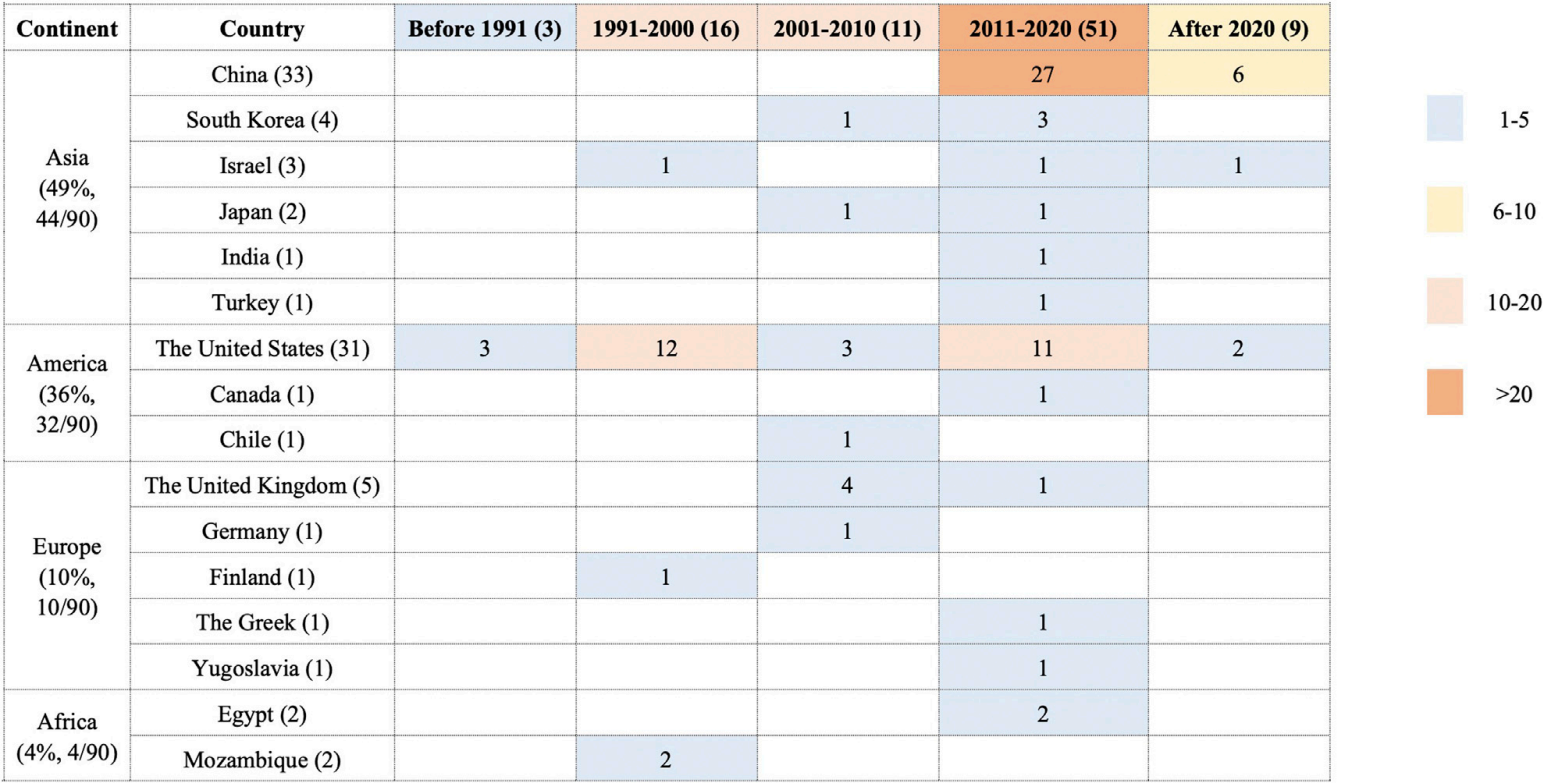
The published areas and time of the including studies.

The search retrieved 6,487 studies. After removing duplicates and irrelevant records by screening the titles and abstracts, 230 studies were assessed for eligibility by full-text screening. Eventually, 90 studies (Chatzakis et al., 2020) were included in this systematic review (Figure 1). These studies were conducted in 17 countries on five continents from 1966 to 2021 (Figure 2). The study designs were comprised of systematic review (7/90, 7.8%) (Mercer and Arheart, 1995; Maymon et al., 1998; Kenyon et al., 2004; Cousens et al., 2010; Wojcieszek et al., 2014; Saccone and Berghella, 2015; Chatzakis et al., 2020), RCTs (32/90, 35.6%) (Brelje and Kaltreider, 1966; Amon et al., 1988; Johnston et al., 1990; McGregor et al., 1991; Kurki et al., 1992; McCaul et al., 1992; Mercer et al., 1992; Lockwood et al., 1993; Ernest and Givner, 1994; Lewis et al., 1995; Almeida et al., 1996; Grable et al., 1996; Lovett et al., 1997; Kenyon et al., 2001b; Ovalle et al., 2002; Lewis et al., 2003; Segel et al., 2003; August Fuhr et al., 2006; Kwak et al., 2013; Nabhan et al., 2014; Zhang, 2014; Kahramanoglu et al., 2016; Mai and He, 2016; Liang, 2018; Zheng, 2018; Pasquier et al., 2019; Siegel et al., 2019; Deng, 2020; Wolf et al., 2020; Chen, 2021; Cong, 2021; Zheng, 2021) and cohort studies (51/90, 56.6%) (A, 2021; Ali, 2020; Bar et al., 2020; Barišić et al., 2017; Bergström, 1991; Chang et al., 2017; Chen et al., 2020; Dotters-Katz et al., 2017; Du, 2016; Du et al., 2019; Du and Zhang, 2020; Ehsanipoor et al., 2008; Feng, 2020; Finneran et al., 2019; Finneran et al., 2017; Fitzgibbon et al., 2021; Siegel et al., 2019; Ke, 2013; Kenyon et al., 2008; Knupp et al., 2022; Kole-White et al., 2021; Lee et al., 2016; Li, 2017; Li, 2020; Lin et al., 2012; Martingano et al., 2020; Navathe et al., 2019; Pan et al., 2018; Pawar and Reddy, 2020; Pierson et al., 2014; Edwards et al., 2020; Ryo et al., 2005; Smith et al., 2015; Song and Han, 2005; Sung et al., 2017; Tai, 2011; Tanaka et al., 2019; Kramer et al., 1996; Wu, 2018; Yeung et al., 2014; Zeng and Lin, 2020; Zhang, 2017; Zhang, 2019;Zhao, 2019a; Zhao,2019b; Zheng et al., 2016; Zhou et al., 2015; Zhou, 2020; Zou, 2021; Zheng et al., 2020). Out of the 90 studies, 78 (86.7%) studies (Ali, 2020; Almeida et al., 1996; Amon et al., 1988; Bar et al., 2000; Bergström, 1991; Chang et al., 2017; Chatzakis et al., 2020; Chen et al., 2020; Chen, 2021; Cong, 2021; Cousens et al., 2010; Lewis et al., 1995; Deng, 2020; Dotters-Katz et al., 2017; Du, 2016; Du et al., 2019; Du and Zhang, 2020; Ehsanipoor et al., 2008; Ernest and Givner, 1994; Feng, 2020; Finneran et al., 2019; Finneran et al., 2017; Fitzgibbon et al., 2021; August et al., 2006; Grable et al., 1996; Siegel et al., 2019; Johnston et al., 1990; Kahramanoglu et al., 2016; Ke, 2013; Kenyon et al., 2001; Kenyon et al., 2004; Knupp et al., 2022; Kole-White et al., 2021; Kurki et al., 1992; Kwak et al., 2013; Lee et al., 2016; Li, 2017; Li, 2020; Li ,2021; Liang, 2018; Lin et al., 2012; Lockwood et al., 1993; Lovett et al., 1997; Siegel et al., 2019; Mai et al., 2016; Martingano et al., 2020; Maymon et al., 1998; McCaul et al., 1992; McGregor et al., 1991; Mercer et al., 1992; Mercer and Arheart, 1995; Lewis et al., 2003; Nabhan et al., 2014; Navathe et al., 2019; Ovalle et al., 2002; Pan et al., 2018; Pasquier et al., 2019; Pawar and Reddy, 2020; Pierson et al., 2014; Edwards et al., 2020; Ryo et al., 2005; Saccone and Berghella, 2015; Segel et al., 2003; Smith et al., 2015; Song et al. 2005; Sung et al., 2017; Tai, 2011; Tanaka et al., 2019; Kramer et al., 1996; Wojcieszek et al., 2014; Wolf et al., 2020; Wu, 2018; Yeung et al., 2014; Zeng and Lin, 2020; Zhang, 2014; Zhang, 2017; Zhang, 2019; Zhao, 2019a; Zhao, 2019b; Zheng et al., 2016; Zheng, 2021; Zhou et al., 2015; Zhou, 2020; Zou, 2021) included PPROM women, 6 (6.7%) studies (Zheng, 2018; A, 2021; Barišić et al., 2017; Tai, 2011; Zhao, 2019a; Zheng et al., 2020) included term PROM women, 4 (4.4%) studies (Kwak et al., 2013; Nabhan et al., 2014; Wojcieszek et al., 2014; Saccone and Berghella, 2015) included both PPROM and term PROM women and 2 (2.2%) studies (Brelje and Kaltreider, 1966; Kenyon et al., 2008) did not report whether the participants were term. The study interventions/comparisons included: 1) using antibiotics vs placebo/blank control (31/90, 34.4%) (Brelje and Kaltreider, 1966; Amon et al., 1988; Johnston et al., 1990; Bergström, 1991; Kurki et al., 1992; McCaul et al., 1992; Mercer et al., 1992; Lockwood et al., 1993; Ernest and Givner, 1994; Mercer and Arheart, 1995; Almeida et al., 1996; Grable et al., 1996; Kramer et al., 1996; Maymon et al., 1998; Bar et al., 2020; Ovalle et al., 2002; Kenyon et al., 2004; Song and Han, 2005; August Fuhr et al., 2006; Cousens et al., 2010; Lin et al., 2012; Ke, 2013; Nabhan et al., 2014; Wojcieszek et al., 2014; Zhang, 2014; Saccone and Berghella, 2015; Du, 2016; Chang et al., 2017; Dotters-Katz et al., 2017; Pasquier et al., 2019; Feng, 2020); 2) different antibiotics (29/90, 32.2%) (McGregor et al., 1991; Lewis et al., 1995; Lovett et al., 1997; Edwards et al., 2020; Kenyon et al., 2001b; Ryo et al., 2005; Ehsanipoor et al., 2008; Kenyon et al., 2008; Kwak et al., 2013; Pierson et al., 2014; Yeung et al., 2014; Kahramanoglu et al., 2016; Lee et al., 2016; Zheng et al., 2016; Finneran et al., 2017; Sung et al., 2017; Wu, 2018; Zhao, 2019b; Finneran et al., 2019; Navathe et al., 2019; Siegel et al., 2019; Tanaka et al., 2019; Ali, 2020; Chatzakis et al., 2020; Martingano et al., 2020; Pawar and Reddy, 2020; Wolf et al., 2020; Fitzgibbon et al., 2021); 3) different timing of antibiotics administration (17/90, 18.9%) (Deng, 2020; Liang, 2018; Zheng, 2018; A, 2021; Barišić et al., 2017; Tai, 2011; Zhao, 2019a; Zheng et al., 2020; Chen et al., 2020; Du et al., 2019; Du and Zhang, 2020; Knupp et al., 2022; Li, 2017; Pan et al., 2018; Zeng and Lin, 2020; Zhang, 2019; Zhou et al., 2015); 4) antibiotics chosen depending on experience vs culture results (8/90, 8.9%) (Mai and He, 2016; Zhang, 2017; Zhou, 2020; Chen, 2021; Cong, 2021; Zheng, 2021; Zou, 2021); 5) different courses of antibiotics administration (4/90, 4.4%) (Lewis et al., 2003; Segel et al., 2003; Smith et al., 2015; Li, 2020); 6) different administration route (1/90, 1.1%) (Kole-White et al., 2021). The median number of the outcomes in the included studies was 7, with the range 1–31. Only 38.9% (35/90) studies (Chatzakis et al., 2020; Saccone and Berghella, 2015; Amon et al., 1988; Brelje and Kaltreider, 1966; Lewis et al., 1995; Ernest and Givner, 1994; Grable et al., 1996; Siegel et al., 2019; Kahramanoglu et al., 2016; Kenyon et al., 2001b; Kurki et al., 1992; Kwak et al., 2013; Lockwood et al., 1993; Mercer et al., 1992; Nabhan et al., 2014; Pasquier et al., 2019; Segel et al., 2003; A, 2021; Zhao, 2019a; Zheng et al., 2020; Kenyon et al., 2008; Bar et al., 2020; Chang et al., 2017; Dotters-Katz et al., 2017; Kramer et al., 1996; Ehsanipoor et al., 2008; Fitzgibbon et al., 2021; Martingano et al., 2020; Pierson et al., 2014; Sung et al., 2017; Zheng et al., 2016; Knupp et al., 2022; Smith et al., 2015; Kole-White et al., 2021) defined study outcomes and 3.3% (3/90) studies (Kenyon et al., 2008; Kwak et al., 2013; Chang et al., 2017) explained how to measure the outcomes. 16.7% (15/90) of studies used composite outcomes (Lockwood et al., 1993; Kenyon et al., 2001b; Segel et al., 2003; Kenyon et al., 2008; Kwak et al., 2013; Wojcieszek et al., 2014; Smith et al., 2015; Kahramanoglu et al., 2016; Zheng et al., 2016; Chang et al., 2017; Zhao, 2019a; Pasquier et al., 2019; Siegel et al., 2019; Zheng et al., 2020; Knupp et al., 2022). Supplementary Table S2 shows the study characteristics.

#### 3.1.2 Outcomes Reported in the Studies

Extraction of each verbatim outcome domain from each study, a total of 784 verbatim outcomes were identified. After merging outcomes with similar definitions and removing duplicates, we had 109 unique outcomes. Of those, 76.1% (83/109) of outcomes were not clearly defined and often had different definitions for the same term. For example, the definition of “latency period” was provided in 11 studies (Lockwood et al., 1993; Ernest and Givner, 1994; Grable et al., 1996; Pierson et al., 2014; Smith et al., 2015; Chang et al., 2017; Dotters-Katz et al., 2017; Sung et al., 2017; Siegel et al., 2019; Fitzgibbon et al., 2021; Kole-White et al., 2021); however, some studies meant “time from the first dose of antibiotics to delivery” (Pierson et al., 2014; Sung et al., 2017; Kole-White et al., 2021) and other studies meant “from the day of rupture of membranes to the date of delivery” (Lockwood et al., 1993; Ernest and Givner, 1994; Grable et al., 1996; Smith et al., 2015; Chang et al., 2017; Dotters-Katz et al., 2017; Siegel et al., 2019; Fitzgibbon et al., 2021).

Since the antibiotics strategy dramatically differs between PPROM and TPROM, we analyzed these subsets of pregnancy complications separately. Outcomes were categorized according to the populations in the studies reporting these outcomes, with PPROM having more outcomes than TPROM, 97 and 70, respectively.

**FIGURE 3:**
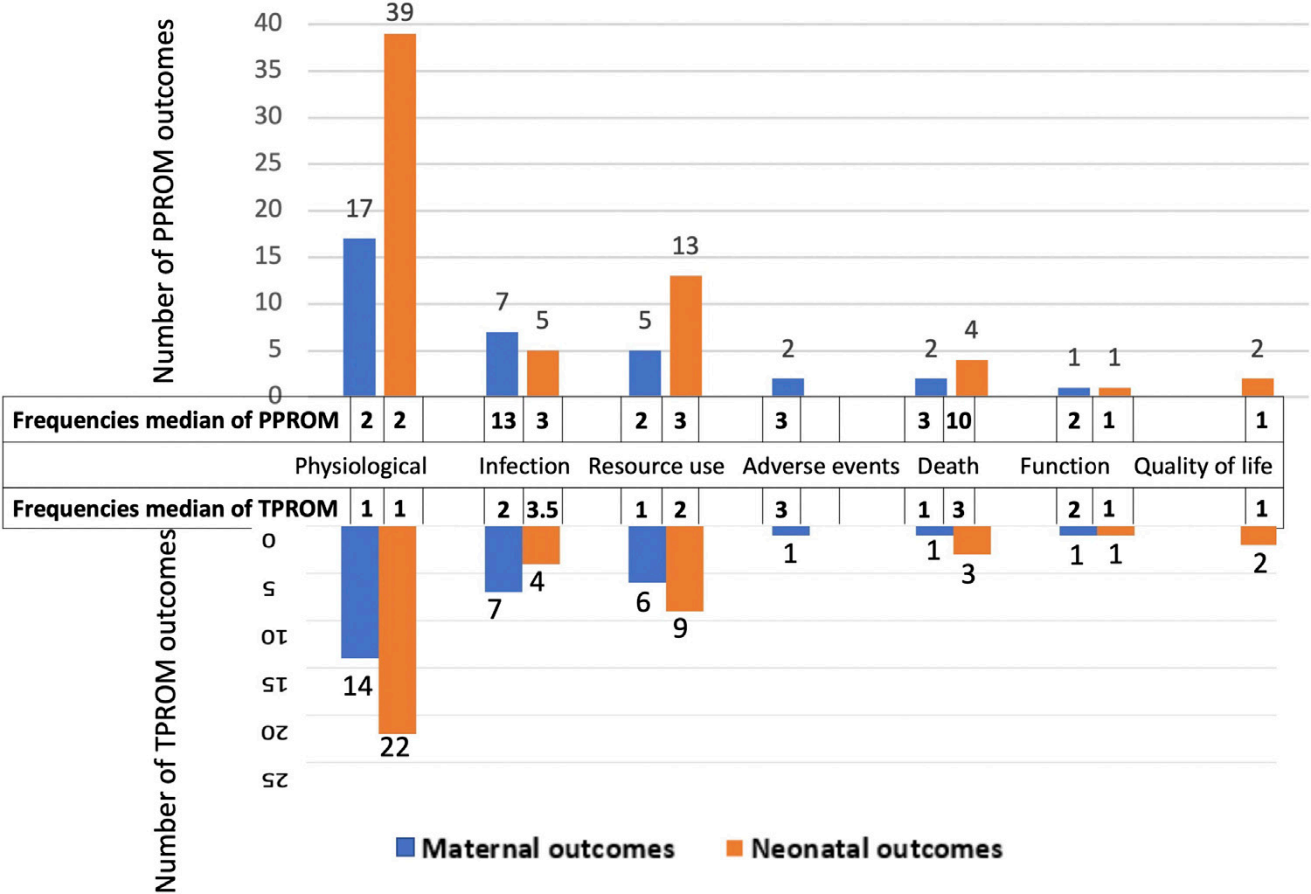
Summary of core outcome areas.

The 97 outcomes for PPROM were grouped into maternal outcomes and neonatal outcomes. Maternal outcomes involved 33 outcomes categorized into six core domains (physiological, infection, resource use, death, adverse events, and function, from most to least). Neonatal outcomes involved 64 outcomes categorized into six core domains (physiological, resource use, infection, death, quality of life, and function, from most to least) (Figure 3). The physiological domain was the most common for maternal and neonatal outcomes, with the 51.5% (17/33) and 60.9% (39/64) outcomes falling into it, respectively.

Table 1 presents outcomes for PPROM with the number of reported studies (reported frequencies). Figure 3 ranks the outcome domains by median reported frequencies from high to low. The rank for maternal outcome domains were infection, death, adverse events, physiological, function and resource use, and for neonatal domains were death, infection, resource use, physiological, quality of life, and function. Across all maternal outcomes, the top three most frequently reported outcomes were chorioamnionitis, pregnancy latency period, and mode of delivery, reported in 47.8% (43/90), 45.6% (41/90), and 30.0% (27/90), respectively of the including studies. The top three most frequently reported outcomes for newborns were neonatal sepsis, neonatal deaths, and birth weight, reported in 38.9% (35/90), 37.8% (34/90), and 36.7% (33/90) of the included studies. Nevertheless, 35.1% of outcomes (34/97, eight maternal and 26 neonatal outcomes) were reported only once in the related studies.

**TABLE 1:** The initial outcomes list of COS for antibiotics in PROM.

Outcome domain	Outcome	Number of reported studies	Definition	Participants’ views
PROM (97)				
Physiological (17/97)	Latency period	41 (Johnston et al. (1990); Bergström, (1991); McGregor et al. (1991); McCaul et al. (1992); Lockwood et al. (1993); Ernest and Givner, 1994; Lewis et al. (1995); Mercer and Arheart, (1995); Almeida et al. (1996); Grable et al. (1996); Lovett et al. (1997); Maymon et al. (1998); Bar et al. (2020); Kenyon et al. (2001b); Lewis et al. (2003); Segel et al. (2003); Kenyon et al. (2004); Ryo et al. (2005); August Fuhr et al. (2006); Pierson et al. (2014); Saccone and Berghella, (2015); Smith et al. (2015); Kahramanoglu et al. (2016); Mai and He, (2016); Chang et al. (2017); Dotters-Katz et al. (2017); Finneran et al. (2017); Sung et al. (2017); Wu, (2018); Du et al. (2019); Navathe et al. (2019); Siegel et al. (2019); Zhang, (2019); Chatzakis et al. (2020); Du and Zhang, (2020); Martingano et al. (2020); Pawar and Reddy, (2020); Zeng and Lin, (2020); Fitzgibbon et al. (2021); Kole-White et al. (2021); Knupp et al. (2022))	√ (Lockwood et al. (1993); Ernest and Givner, (1994); Grable et al. (1996); Pierson et al. (2014); Smith et al. (2015); Chang et al. (2017); Dotters-Katz et al. (2017); Sung et al. (2017); Siegel et al. (2019); Fitzgibbon et al. (2021); Kole-White et al. (2021))	√
	Mode of delivery	27 Brelje and Kaltreider, (1966); Johnston et al., 1990; Lewis et al. (1995); Grable et al. (1996); Bar et al. (2020); Kenyon et al. (2001b); Ke, (2013); Lee et al. (2016); Finneran et al. (2017); Ali, (2020); Feng, (2020); Chen, (2021); Fitzgibbon et al. (2021)	—	√
	Postpartum hemorrhage	22 Lin et al. (2012); Nabhan et al. (2014); Wojcieszek et al. (2014); Zhou et al. (2015); Li, (2017); Zhang, (2017); Pan et al. (2018); Zhao, (2019b); Du et al. (2019); Chen et al. (2020); Deng, (2020); Du and Zhang, (2020); Feng, (2020); Li, (2020); Zeng and Lin, (2020); Zhou, (2020); Chen, (2021); Cong, (2021); Zheng, (2021); Zou, (2021); Knupp et al. (2022))	—	—
	Preterm delivery	6 Brelje and Kaltreider, (1966); Lee et al. (2016); Feng, (2020)	—	—
	Maternal white blood cell count	4 (Kahramanoglu et al. (2016); Liang, (2018); Wu, (2018); Fitzgibbon et al. (2021))	—	—
	Placental abruption	4 (Mercer et al. (1992); Lewis et al. (1995); Saccone and Berghella, (2015); Pawar and Reddy, (2020)	—	—
	Deep vein thrombosis	3 Dotters-Katz et al. (2017); Ali (2020); Knupp et al. (2022)	√ (Dotters-Katz et al. (2017))	—
	Maternal c-reactive protein	3 Kahramanoglu et al. (2016); Liang, (2018); Wu, (2018)	—	—
	Fever	2 Wojcieszek et al. (2014); Kahramanoglu et al. (2016)	√ Wojcieszek et al. (2014)	—
	Maternal intensive care unit admission	2 Wojcieszek et al. (2014); Fitzgibbon et al. (2021)	—	—
	Meconium-stained amniotic fluid	2 Feng, (2020); Martingano et al. (2020)	—	√
	Amniotic fluid index	2 (Lewis et al. (1995); Kahramanoglu et al. (2016))	—	√
	Cardiac arrest	1 (Wojcieszek et al. (2014))	—	—
	Cord prolapse	1 Saccone and Berghella, (2015)	—	—
	Reason for delivery	1 Finneran et al. (2017)	—	—
	Respiratory arrest	1 Wojcieszek et al. (2014)	—	—
	Trophoblastic hyperplasia	1 Ovalle et al. (2002)	—	—
Infection (7/97)	Chorioamnionitis	43 Brelje and Kaltreider, (1966); Amon et al. (1988); Johnston et al. (1990); Kurki et al. (1992); Grable et al. (1996); Bar et al. (2020); Kenyon et al. (2004); Ehsanipoor et al. (2008); Du, (2016); Lee et al. (2016); Siegel et al. (2019); Ali (2020); Chatzakis et al. (2020); Deng, (2020); Chen, (2021); Cong, (2021); Knupp et al. (2022))	√ (Amon et al. (1988); Kurki et al. (1992); Mercer et al. (1992); Grable et al. (1996); Ehsanipoor et al. (2008); Wojcieszek et al. (2014); Zheng et al. (2016); Pasquier et al. (2019); Martingano et al. (2020))	—
	Endometritis	18 Brelje and Kaltreider, (1966); Amon et al. (1988); Johnston et al. (1990); Kurki et al. (1992); Mercer et al. (1992); Ernest and Givner, (1994); Grable et al. (1996); Kramer et al. (1996); Maymon et al. (1998); Edwards et al. (2020); Segel et al. (2003); Ehsanipoor et al. (2008); Nabhan et al. (2014); Wojcieszek et al. (2014); Saccone and Berghella, (2015); Martingano et al. (2020); Knupp et al. (2022)	√ Amon et al. (1988); Mercer et al. (1992); Ernest and Givner, (1994); Grable et al. (1996); Kramer et al. (1996); Ehsanipoor et al. (2008); Wojcieszek et al. (2014); Martingano et al. (2020)	
	Puerperal infection	18 Mercer and Arheart, (1995); Zhou et al. (2015); Li, (2017); Zhang, (2017); Pan et al. (2018); Zhao, (2019b); Du et al. (2019); Ali (2020); Chen et al. (2020); Du and Zhang, (2020); Feng, (2020); Li, (2020); Zeng and Lin, (2020); Zhou, (2020); Chen, (2021); Cong, (2021); Zheng, (2021); Zou, (2021)	—	—
	Intrauterine infection	13 Ernest and Givner, (1994); Ovalle et al. (2002); Zhou et al. (2015); Du, (2016); Finneran et al. (2017); Li, (2017); Liang, (2018); Wu, (2018); Du et al. (2019); Deng, (2020); Du and Zhang, (2020); Feng, (2020); Li, (2020)	√ Ernest and Givner, (1994)	√
	Maternal sepsis	11 Johnston et al. (1990); Kurki et al. (1992); Mercer et al. (1992); Song and Han, (2005); Wojcieszek et al. (2014); Saccone and Berghella, (2015); Finneran et al. (2017); Sung et al. (2017); Siegel et al. (2019); Pawar and Reddy, (2020); Knupp et al. (2022)	√ Wojcieszek et al. (2014)	—
	Maternal infection	3 (McCaul et al. (1992); Mai and He, (2016); Zhang, (2019)	—	—
	Wound infection	2 Wojcieszek et al. (2014); Knupp et al. (2022)	—	—
Resource use (5/97)	Length of maternal hospitalization	8 (Johnston et al. (1990); Lockwood et al. (1993); Almeida et al. (1996); Lovett et al. (1997); Kenyon et al. (2001b); Nabhan et al. (2014); Wojcieszek et al. (2014); Kahramanoglu et al. (2016))	—	—
	Steroid administration	3 (Bar et al. (2020); Kahramanoglu et al. (2016); Chang et al. (2017))	—	—
	Postpartum antibiotic administration	2 (Kenyon et al. (2001b); Wojcieszek et al. (2014))	—	—
	Tocolysis administration	1 (Kahramanoglu et al. (2016))	—	—
	Cost	1 (Finneran et al. (2019))		√
Adverse events (2/97)	Adverse drug reaction	5 (Nabhan et al. (2014); Pierson et al. (2014); Wojcieszek et al. (2014); Saccone and Berghella, 2015; Sung et al. (2017))	—	—
	Anaphylaxis	1 (Wojcieszek et al. (2014))	—	—
Death (2/97)	Maternal deaths	3 (Wojcieszek et al. (2014); Zhang, (2017); Knupp et al. (2022))	—	—
Function (1/97)	Breastfeeding	2 (Wojcieszek et al. (2014); Saccone and Berghella, (2015)	—	—
Physiological (39/97)	Birth weight	33 Johnston et al. (1990); Bergström, (1991); Ernest and Givner, (1994); Lewis et al. (1995); Almeida et al. (1996); Grable et al. (1996); Bar et al. (2020); Kenyon et al. (2001b); Kwak et al. (2013); Du, 2016; Kahramanoglu et al. (2016); Lee et al. (2016); Chang et al. (2017); Chen, (2021); Knupp et al. (2022)	—	√
	Respiratory distress syndrome	32 Johnston et al. (1990); Lewis et al. (1995); Grable et al. (1996); Bar et al. (2020); Kenyon et al. (2001b); August Fuhr et al. (2006); Ehsanipoor et al. (2008); Cousens et al. (2010); Ke, (2013); Kwak et al. (2013); Kahramanoglu et al. (2016); Lee et al. (2016); Chang et al. (2017); Finneran et al. (2017); Chatzakis et al. (2020); Knupp et al. (2022)	√ Grable et al. (1996); Ehsanipoor et al. (2008)	—
	Apgar score	30 Brelje and Kaltreider, (1966); Johnston et al. (1990); Kurki et al. (1992); Mercer et al. (1992); Lockwood et al. (1993); Grable et al. (1996); Lovett et al. (1997); Bar et al. (2020); Lewis et al. (2003); Kwak et al. (2013); Nabhan et al. (2014); Pierson et al. (2014); Wojcieszek et al. (2014); Saccone and Berghella, 2015; Zhou et al. (2015); Du, (2016); Kahramanoglu et al. (2016); Finneran et al. (2017); Li, (2017); Sung et al. (2017); Wu, (2018); Du et al. (2019); Navathe et al. (2019); Tanaka et al. (2019); Zhang, (2019); Du and Zhang, (2020); Li, (2020); Wolf et al. (2020); Zeng and Lin, (2020); Fitzgibbon et al. (2021)		
	Necrotising enterocolitis	27 (Johnston et al. (1990); Grable et al. (1996); Bar et al. (2020); Kenyon et al. (2001b); Segel et al. (2003); August Fuhr et al. (2006); Ehsanipoor et al. (2008); Cousens et al. (2010); Kwak et al. (2013); Lee et al. (2016); Chang et al. (2017); Finneran et al. (2017); Pasquier et al. (2019); Siegel et al. (2019); Chatzakis et al. (2020); Knupp et al. (2022))	√ Grable et al. (1996); Kenyon et al. (2001b); Ehsanipoor et al. (2008); Siegel et al. (2019)	
	Neonatal pneumonia	19 McGregor et al. (1991); Mercer et al. (1992); Mercer and Arheart, (1995); Ehsanipoor et al. (2008); Lin et al. (2012); Ke, (2013); Nabhan et al. (2014); Wojcieszek et al. (2014); Zhang, (2014); Smith et al. (2015); Kahramanoglu et al. (2016); Zhang, (2017); Zhao, (2019b); Feng, (2020); Zeng and Lin, (2020); Zhou, (2020); Chen, (2021); Zheng, (2021); Zou, (2021)	√ Ehsanipoor et al. (2008)	—
	Neonatal infection	15 Brelje and Kaltreider, (1966); Amon et al. (1988); Ernest and Givner, (1994); Lewis et al. (1995); Bar et al. (2020); Kenyon et al. (2004); August Fuhr et al. (2006); Saccone and Berghella, (2015); Smith et al. (2015); Du, (2016); Chang et al. (2017); Chatzakis et al. (2020); Feng, (2020); Wolf et al. (2020); Zeng and Lin, (2020)	√ Brelje and Kaltreider, (1966); Amon et al. (1988); Lewis et al. (1995); Bar et al. (2020); Smith et al. (2015)	
	Bronchopulmonary dysplasia	11 Kurki et al. (1992); Ehsanipoor et al. (2008); Kwak et al. (2013); Lee et al. (2016); Chang et al. (2017); Siegel et al. (2019); Knupp et al. (2022)	√ Ehsanipoor et al. (2008)	—
	Neonatal asphyxia	9 Ke, (2013); Zhang, (2014); Liang, (2018); Pan et al. (2018); Zhao, (2019b); Zhang, (2019); Chen et al. (2020); Du and Zhang, (2020); Feng, (2020)	—	—
	Periventricular leukomalacia	9 Lee et al. (2016); Chang et al. (2017); Siegel et al. (2019)	—	—
	Cerebral palsy	7 Kenyon et al. (2008); Lee et al. (2016); Siegel et al. (2019)	—	—
	Fetal distress	7 (Mercer et al. (1992); Lin et al. (2012); Pan et al. (2018); Zhao, 2019b; Chen et al. (2020); Du and Zhang, 2020; Feng, 2020)	—	√
	Cord arterial pH	4 (Johnston et al. (1990); Lockwood et al. (1993); Grable et al. (1996); Wolf et al. (2020))	—	—
	Neonatal icterus	4 Lin et al. (2012); Kahramanoglu et al. (2016); Pan et al. (2018); Feng, (2020)	—	—
	Retinopathy of prematurity	4 Song and Han, (2005); Kwak et al. (2013); Chang et al. (2017)	—	—
	Abnormal brain sonography	3 Kenyon et al. (2004); Kwak et al. (2013); Saccone and Berghella, (2015)	√ Saccone and Berghella, (2015)	—
	Neonatal fever	2 Smith et al. (2015); Knupp et al. (2022)	—	—
	Neurological outcome	2 Kwak et al. (2013); Chang et al. (2017)	√ Chang et al. (2017)	—
	Patent ductus arteriosus	2 Lewis et al. (1995); Tanaka et al. (2019)	—	—
	Respiratory problems	2 Kenyon et al. (2008); Saccone and Berghella, (2015)	√ Kenyon et al. (2008)	—
	Seizures	2 Kenyon et al. (2008); Knupp et al. (2022)	—	—
	Small for gestational age	2 Johnston et al. (1990); McGregor et al. (1991)	—	—
	Abnormal hearing screen	1 Tanaka et al. (2019)	—	—
	Bowel disorders	1 Kenyon et al. (2008)	—	—
	Chronic lung disease	1 Kenyon et al. (2001b)	—	—
	Conjunctivitis	1 McGregor et al. (1991)	—	—
	Diabetes	1 Kenyon et al. (2008)	—	—
	Fetal placental vascular lesions	1 Ovalle et al. (2002)	—	—
	Hypoxic ischemic encephalopathy	1 Zhang, (2014)	—	—
	Neonatal group B *streptococcus* colonization	1 Yeung et al. (2014)	—	—
	Neonatal group B *streptococcus* infection	1 Yeung et al. (2014)	—	—
	Neonatal scleredema	1 Zhang, (2014)	—	—
	neonatal white cell count	1 Fitzgibbon et al. (2021)	—	—
	Patent ductus arteriosus ligated	1 Tanaka et al. (2019)	—	—
	Persistent fetal circulation	1 Grable et al. (1996)	√ Grable et al. (1996)	—
	Postnatal steroid requirement	1 Tanaka et al. (2019)	—	—
	Pulmonary hypoplasia	1 Knupp et al. (2022)	—	—
	Skeletal deformities	1 Kurki et al. (1992)	—	—
	Transient tachypnea of the newborn	1 Kahramanoglu et al. (2016)	—	—
	Weight gain	1 Johnston et al. (1990)	—	—
Resource use (13/97)	Admission to the neonatal intensive care unit	9 Lewis et al. (1995); Kenyon et al. (2001b); Lewis et al. (2003); Kwak et al. (2013); Nabhan et al. (2014); Wojcieszek et al. (2014); Saccone and Berghella, (2015); Kahramanoglu et al. (2016); Chatzakis et al. (2020)	—	—
	Duration of hospitalization of the newborns	9 McCaul et al. (1992); Mercer et al. (1992); Almeida et al. (1996); Wojcieszek et al. (2014); Saccone and Berghella, (2015); Liang, 2018; Navathe et al. (2019); Tanaka et al. (2019); Wolf et al. (2020)	—	—
	Duration of stay in the neonatal intensive care unit	7 Johnston et al. (1990); Lockwood et al. (1993); Kwak et al. (2013); Nabhan et al. (2014); Wojcieszek et al. (2014); Finneran et al. (2017); Knupp et al. (2022)	—	—
	Duration of ventilation	5 Lewis et al. (1995); Lovett et al. (1997); Kwak et al. (2013); Nabhan et al. (2014); Tanaka et al. (2019)	—	—
	Mechanical ventilation requirement	5 Kurki et al. (1992); Lovett et al. (1997); Kenyon et al. (2001b); Kwak et al. (2013); Wojcieszek et al. (2014)	—	—
	Oxygen requirement	4 Lewis et al. (1995); Lovett et al. (1997); Kenyon et al. (2001b); Pasquier et al. (2019)	—	—
	Antibiotic therapy requirement	3 Wojcieszek et al. (2014); Saccone and Berghella, 2015; Wolf et al. (2020)	—	—
	Hospital admission	3 McGregor et al. (1991); Lewis et al. (1995); Kenyon et al. (2008)	—	—
	Duration of antibiotics	2 Johnston et al. (1990); Tanaka et al. (2019)	—	—
	Duration of oxygen requirement	2 Lewis et al. (1995); Lovett et al. (1997)	—	—
	Surfactant requirement	2 Kenyon et al. (2001b); Tanaka et al. (2019)	—	—
	Internal fetal monitoring	1 Wojcieszek et al. (2014)	—	—
	Neonatal respiratory support	1 Wolf et al. (2020)	—	—
Infection (5/97)	Neonatal sepsis	35 Johnston et al. (1990); Kurki et al. (1992); Kenyon et al. (2001b); Cousens et al. (2010); Kwak et al. (2013); Kahramanoglu et al. (2016); Lee et al. (2016); Chang et al. (2017); Siegel et al. (2019); Chen et al. (2020); Knupp et al. (2022)	√ (Kramer et al. (1996); Nabhan et al. (2014); Wojcieszek et al. (2014); Saccone and Berghella, (2015); Martingano et al. (2020)	—
	Intraventricular haemorrhage	26 Johnston et al. (1990); Lewis et al. (1995); Grable et al. (1996); Bar et al. (2020); August Fuhr et al. (2006); Cousens et al. (2010); Kwak et al. (2013); Lee et al. (2016); Chang et al. (2017); Siegel et al. (2019); Chatzakis et al. (2020); Knupp et al. (2022)	√ Grable et al. (1996); Siegel et al. (2019)	—
	Funisitis	3 Lee et al. (2016)	√ Zheng et al. (2016)	—
	Neonatal meningitis	1 Wojcieszek et al. (2014)	—	—
	Intracranial infection	1 Zeng and Lin, (2020)	—	—
Death (4/97)	Neonatal deaths	34 Brelje and Kaltreider, (1966); Johnston et al. (1990); Bergström, (1991); Kurki et al. (1992); Bar et al. (2020); Kenyon et al. (2001b); Kenyon et al. (2008); Cousens et al. (2010); Ke, (2013); Kwak et al. (2013); Kahramanoglu et al. (2016); Lee et al. (2016); Dotters-Katz et al. (2017); Finneran et al. (2017); Siegel et al. (2019); Chatzakis et al. (2020); Knupp et al. (2022)	—	√
	Perinatal death	10 McGregor et al. (1991); Kurki et al. (1992); Maymon et al. (1998); Lewis et al. (2003); Kenyon et al. (2004); Nabhan et al. (2014); Wojcieszek et al. (2014); Saccone and Berghella, 2015; Zhao, (2019b); Chatzakis et al. (2020)	√ Chatzakis et al. (2020)	—
	Stillbirth	5 Johnston et al. (1990); Bergström, (1991); Kurki et al. (1992); Mercer et al. (1992); Wojcieszek et al. (2014)	—	—
	Neonatal deaths due to infection	1 Mercer et al. (1992)	—	—
Quality of life (2/97)	Health-related quality-of-life and behavior	1 Kenyon et al. (2008)	—	√
	Developmental problems	1 Kenyon et al. (2008)	√ Kenyon et al. (2008)	
Function (1/97)	Functional impairment	1 Kenyon et al. (2008)	—	—
TPROM (70)				
Physiological (14/70)	Mode of delivery	5 Brelje and Kaltreider, (1966); Tai, (2011); Nabhan et al. (2014); Saccone and Berghella, (2015)	—	√
	Postpartum hemorrhage	5 Wojcieszek et al. (2014); Nabhan et al. (2014); A, (2021); Tai, (2011); Zheng et al. (2020)	√ A, (2021)	—
	Latency period	2 Saccone and Berghella, (2015); Barišić et al. (2017)	—	√
	Preterm delivery	2 Brelje and Kaltreider, (1966); Saccone and Berghella, (2015)	—	—
	Temperature	2 Zhao, (2019a); Zheng et.al. (2016); Zheng, (2018); Zheng et.al. (2020)	—	—
	Abnormalities in blood routine	1 Zhao, (2019a)	—	—
	Maternal neutrophil percentage	1 Zheng et al. (2020)	—	—
	Maternal procalcitonin	1 Zheng et al. (2020)	—	—
	Maternal white blood cell count	1 Zheng et al. (2020)	—	—
	Maternal c-reactive protein	1 Zheng et al. (2020)	—	—
	Cord prolapse	1 Saccone and Berghella, (2015)	—	—
	Fever	1 Wojcieszek et al. (2014)	√ Wojcieszek et al. (2014)	—
	Placental abruption	1 Saccone and Berghella, (2015)	—	—
	Respiratory arrest	1 (Wojcieszek et al. (2014))	—	—
Infection (7/70)	Chorioamnionitis	8 Saccone and Berghella, (2015); Wojcieszek et al. (2014); Brelje and Kaltreider, (1966); Nabhan et al. (2014); Zheng, (2018); A, (2021); Barišić et al. (2017); Zheng et al. (2020)	√ Wojcieszek et al. (2014); A, (2021)	—
	Endometritis	4 Brelje and Kaltreider, (1966); Nabhan et al. (2014); Wojcieszek et al. (2014); Saccone and Berghella, (2015)	√ Wojcieszek et al. (2014)	—
	Puerperal infection	3 A, (2021); Tai, (2011); Zheng et al. (2020)	√ A, (2021)	—
	Maternal sepsis	2 Wojcieszek et al. (2014); Saccone and Berghella, (2015)	√ Wojcieszek et al. (2014)	—
	Wound infection	2 Wojcieszek et al. (2014); Wolf et.al. (2020); Wu, (2018); Yeung et.al. (2014); Zeng and Lin, (2020); Zhang, (2014); Zhang, (2017); Zhang, (2019); Zhoa, (2019a); Zhoa, (2019b)	—	—
	Urinary tract infection	1 Zheng et al. (2020)	—	—
	Vaginitis	1 Zheng et al. (2020)	—	—
Resource use (6/70)	Length of maternal hospitalization	2 Nabhan et al. (2014); Wojcieszek et al. (2014)	—	—
	Maternal intensive care unit admission	1 Wojcieszek et al. (2014)	—	—
	Postpartum antibiotic administration	1 Wojcieszek et al. (2014)	—	—
	Anaphylaxis	1 Wojcieszek et al. (2014)	—	—
	Cardiac arrest	1 Wojcieszek et al. (2014)	—	—
Death (1/70)	Maternal deaths	1 Wojcieszek et al. (2014)	—	—
Adverse events (1/70)	Adverse drug reaction	3 Nabhan et al. (2014); Wojcieszek et al. (2014); Saccone and Berghella, (2015)	—	—
Function (1/70)	Breastfeeding	2 (Wojcieszek et al. (2014); Saccone and Berghella, (2015)	—	—
Physiological (22/70)	Apgar score	6 Saccone and Berghella, (2015); Wojcieszek et al. (2014); Brelje and Kaltreider, (1966); Kwak et al. (2013); Nabhan et al. (2014); A, (2021)	√ A, (2021)	—
	Fetal distress	3 Zheng, (2018); A, (2021); Tai, (2011)	√ A, (2021)	√
	Abnormal brain sonography	2 Kwak et al. (2013); Saccone and Berghella, (2015)	√ Saccone and Berghella, (2015)	—
	Cerebral palsy	2 Kenyon et al. (2008); Saccone and Berghella, (2015)	—	—
	Respiratory distress syndrome	2 (Kwak et al. (2013); Wojcieszek et al. (2014))	—	—
	Respiratory problems	2 Kenyon et al. (2008); Saccone and Berghella, (2015)	√ Kenyon et al. (2008)	—
	Baby gender	1 Kwak et al. (2013)	—	—
	Birth weight	1 Kwak et al. (2013)	—	√
	Bowel disorders	1 Kenyon et al. (2008)	—	—
	Bronchopulmonary dysplasia	1 Kwak et al. (2013)	—	—
	Cord arterial pH	1 Zheng et al. (2020)	—	—
	Diabetes	1 Kenyon et al. (2008)	—	—
	Intraventricular haemorrhage	1 Kwak et al. (2013)	—	—
	Necrotising enterocolitis	1 Kwak et al. (2013)	—	—
	Neonatal asphyxia	1 Zheng et al. (2018)	—	—
	Neonatal c-reactive protein	1 Barišić et al. (2017)	—	—
	Neonatal lung injury	1 Zheng, (2018)	—	—
	Neonatal procalcitonin	1 Zheng et al. (2020)	—	—
	Neonatal white blood cell count	1 Zheng et al. (2020)	—	—
	Neurological outcome	1 Kwak et al. (2013)	—	—
	Retinopathy of prematurity	1 Kwak et al. (2013)	—	—
	Seizures	1 Kwak et al. (2013)	—	—
Resource use (9/70)	Admission to the neonatal intensive care unit	5 Kwak et al. (2013); Nabhan et al. (2014); Wojcieszek et al. (2014); Saccone and Berghella, (2015); Barišić et al. (2017))	—	—
	Antibiotic therapy requirement	3 Wojcieszek et al. (2014); Saccone and Berghella, (2015); Barišić et al. (2017)	—	—
	Duration of hospitalization of the newborns	3 Wojcieszek et al. (2014); Saccone and Berghella, (2015); Barišić et al. (2017)	—	—
	Duration of stay in the neonatal intensive care unit	3 Kwak et al. (2013); Nabhan et al. (2014); Wojcieszek et al. (2014)	—	—
	Hospital admission	2 Kenyon et al. (2008); Knupp et. al. (2022); Kole-White et.al. (2021); Kurki et.al. (1992); Kwak et al. (2013); Lee et.al. (2016)	√ Kenyon et al. (2008)	—
	Mechanical ventilation requirement	2 Kwak et al. (2013); Wojcieszek et al. (2014)	—	—
	Duration of ventilation	1 Nabhan et al. (2014)	—	—
	Duration of ventilator treatment	1 Kwak et al. (2013)	—	—
	Internal fetal monitoring	1 Wojcieszek et al. (2014)	—	—
Infection (4/70)	Neonatal sepsis	5 Saccone and Berghella, (2015); Wojcieszek et al. (2014); Kwak et al. (2013); Nabhan et al. (2014); A, (2021)	√ Saccone and Berghella, (2015); Wojcieszek et al. (2014); Nabhan et al. (2014); A, (2021)	—
	Neonatal pneumonia	4 Tai, (2011); Nabhan et al. (2014); Wojcieszek et al. (2014); Zheng, (2018)	—	—
	Neonatal infection	3 Saccone and Berghella, (2015); Brelje and Kaltreider, (1966); A, (2021)	√ Brelje and Kaltreider, (1966); A, (2021)	—
	Neonatal meningitis	1 Wojcieszek et al. (2014)	—	—
Death (3/70)	Neonatal deaths	4 Brelje and Kaltreider, (1966); Kenyon et al. (2008); Kwak et al. (2013); Wojcieszek et al. (2014)	—	√
	Perinatal death	3 Nabhan et al. (2014); Wojcieszek et al. (2014); Saccone and Berghella, (2015)	—	—
	Stillbirth	1 Wojcieszek et al. (2014)	—	—
Quality of life (2/70)	Health-related quality-of-life and behavior	1 Kenyon et al. (2008)	—	√
	Developmental problems	1 Kenyon et al. (2008)	√ Kenyon et al. (2008)	—
Function (1/70)	Functional impairment	1 Kenyon et al. (2008)	—	—

The 70 outcomes for TPROM were divided into maternal outcomes and neonatal outcomes. Maternal outcomes included 29 outcomes and were classified into six core domains, while neonatal outcomes included 41 outcomes classified into six core domains. Besides, the order of domains is the same as for PPROM (Figure 3). The physiological domain was the most common for both maternal and neonatal outcomes, with the 48.3% (14/29) and 53.7% (22/41) outcomes belonging to it, respectively.

Table 1presents the outcomes for TPROM and the number of reported studies. The rank for maternal outcome domains by reported frequencies were adverse events, infection, function, death, physiological, and resource use, and for neonatal domains were infection, death, resource use, physiological, and quality of life (Figure 3). The top three most frequently reported maternal outcomes were chorioamnionitis, postpartum hemorrhage, and mode of delivery, reported in 8.9% (8/90), 5.6% (5/90), and 5.6% (5/90), respectively of the included studies. And the top three most frequently reported neonatal outcomes were Apgar score, neonatal sepsis, and admission to the neonatal intensive care unit, reported in 6.7% (6/90), 5.6% (5/90), and 5.6% (5/90) of the including studies. Nevertheless, 57.1% of outcomes (40/70, 16 maternal and 24 neonatal outcomes) were reported only once in the related studies.

### 3.2 Semi-structured Interview

**FIGURE 4:**
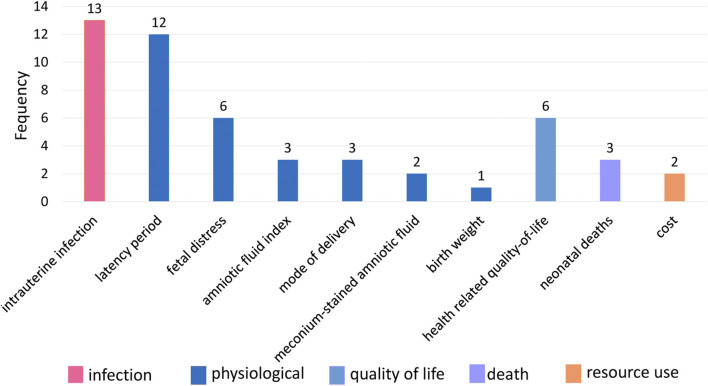
The frequencies of outcomes extracted in the semi-structured interview.

From January 2022 to February 2022, 30 pregnant women took part in the interviews. Their socioeconomic information is in Supplementary Table S3. Two researchers extracted 10 outcomes after normalization, and no new outcomes were obtained (Figure 4). The most frequently reported outcomes by PROM pregnant women were intrauterine infection (43.3%, 13/30), followed by latency period (40.0%, 12/30), fetal distress (20.0%, 6/30), and health-related quality of life and behavior (20.0%, 6/30).

## Discussion

To our knowledge, this is the first study to investigate study outcomes and the concerns of pregnant women on antibiotics in PROM. Our study showed a growing number of studies about antibiotics used in PROM; however, a significant inconsistency appeared in outcomes reported in antibiotics used in pregnant women with PROM. Firstly, the current studies reported many different outcomes, some of which were only reported once. Moreover, many outcomes were not clearly defined, and different definitions were frequently found for the same term. Therefore, it might not be possible to compare, contrast or combine the results of the individual studies in a systematic review to provide higher-level evidence for clinical practice (Clarke and Williamson, 2016), which contributes to waste in research (Glasziou et al., 2014). The development of the COS for antibiotics in PROM could improve the research quality of PROM and provide a reference for research about the infection in pregnant women.

Although the classification and order of the core outcome domains of PPROM and TPROM were consistent, there were some differences between the specific outcomes of PPROM and TPROM studies due to the different clinical stages of PPROM and TPROM. For example, neonatal death was one of the most concerned outcomes of PPROM researchers. However, this outcome was seldom reported in TPROM studies because pre-term birth complications are the leading cause of death among children (World Health Organization, 2018).

The outcomes identified in the including studies could cover the outcomes concerned by pregnant women. The physiological domain contained the most outcomes. Despite this, many outcomes were reported only once in studies or by pregnant women. Both the PPROM studies’ researchers and the pregnant women interviewed were very concerned about the latency period. During the latency period of PROM, the fetus would be exposed to the risk of maternofetal infection, abruptio placentae, cord prolapse, and intrauterine death (Mercer, 2003). However, a large cohort study suggested that prolonged latency duration did not worsen neonatal prognosis. Moreover, survival and survival without severe morbidity improved with increased gestational age at birth (Lorthe et al., 2017). Therefore, prolonging latency if there is no contraindication was recommended in pregnant women at 24 0/7–33 6/7 weeks of gestation (Siegler et al., 2020). Nevertheless, some pregnant women’s concerns, such as health-related quality of life and behavior, were rarely reported in the studies. This kind of outcome is used to assess the effect of chronic disease management on an individual’s health status and is drawing the attention of researchers and policymakers (Guyatt et al., 1993). Although PROM is not a chronic disease, the sequelae of premature infants, according to PROM, require constant attention as many pre-term children develop important behavioral and educational difficulties (Bhutta et al., 2002). Future studies could pay attention to these outcomes.

### Limitation and Future Research

Firstly, our study only included articles in Chinese and English, which could have a language limitation. Besides, the semi-structured interview was conducted at a single center, which could have limitations to sample representativeness. Therefore, in the next stage of this COS research, we would conduct a Delphi survey with stakeholder groups, which were based on multicenter, to add important outcomes not identified by our current study and prioritize outcomes for the COS.

## Conclusion

An initial list of core outcomes set for antibiotics in pregnant women with prelabor rupture of membranes is formed. We identified 109 outcomes from 90 studies and a semi-structured interview. There was considerable inconsistency in outcomes selection and reporting in current studies for antibiotics in PROM. These results provide a robust foundation for the development of a COS.

## Data Availability

The original contributions presented in the study are included in the article/Supplementary Material. Further inquiries can be directed to the corresponding authors.
