# Supplementary material for: Developing a Core Outcome Set for the Evaluation of Antibiotic Use in Prelabor Rupture of Membranes: A Systematic Review and Semi-Structured Interview
Source: Front Pharmacol. 2022 Aug 1;13:915698. doi: 10.3389/fphar.2022.915698 (PMC9376915; doi:10.3389/fphar.2022.915698)
Supplement: Supplementary file 1 [file DataSheet1.docx]

**Supplementary materials**

**Interview Guide**

**Part One: Self-Introductions**

Hi, [participate’s Name]. Thank you for speaking with me today. My name is and I am a researcher at West China Second University Hospital. We are interviewing pregnant women with premature rupture of membranes to assess what kind of outcomes you concerned after you receiving antibiotic treatment for premature rupture of membranes. This information will help us to think about how to best design clinical protocols regarding what patient factors to assess and improving clinical outcomes. All information you provide will be stored on a secure drive accessible only to study team members for the purpose of data analysis. Participation is completely voluntary and you will not be penalized in any way for refusing to participate.

Do you have any questions about the study or the interview before we get started?

May we turn on the tape recorder now?

**Part Two: Opening Questions**

Before we start with the more questions, we’d like to find out a little background regarding your premature rupture of membranes history?

• When did you find out you were premature rupture of membranes?

• Was there much amniotic fluid outflowing after premature rupture of membranes?

PROBE: How many pairs of underwear or pads did you wet?

• How long did you come to the hospital after premature rupture of membranes?

**Part Three: Key Questions**

We are very interested in understanding the factors that you concerned when you developed premature rupture of membranes.

• Were you aware of the risks of premature rupture of membranes during pregnancy?

• What do you know about the risks of premature rupture of membranes?

• After you were hospitalized, did the doctor tell you about the premature rupture of membranes risk? What are the risks?

• What do you feel are/were the outcomes that were most important for you?

PROBE: What are you most worried about? Yourself? Your baby?

**Part Four: Ending Questions**

• Do you know what treatments the doctor provided for you after you were admitted?

• Do you know what benefits of these treatments are for you?

**STable 1 Search terms for each databases**

| **Database** | **Search terms** |
| --- | --- |
| Chinese National Knowledge Infrastructure | (Title/Key words/Abstract= Prelabor rupture of membranes) AND (Title/Key words/Abstract = Antibiotics + Anti-microbial + Penicillins + cephalosporin + azithromycin + clindamycin + erythromycin) |
| Wanfang | Subject headings:( Prelabor rupture of membranes) and Subject headings:( Antibiotics or Anti-microbial or Penicillins or cephalosporin or azithromycin or clindamycin or erythromycin) |
| VIP | (Title/Key words= Prelabor rupture of membranes AND ((((((Title/Key words = Antibiotics OR Title/Key words = Anti-microbial) OR Title/Key words = Penicillins) OR Title/Key words = cephalosporin) OR Title/Key words = azithromycin) OR Title/Key words = clindamycin) OR Title/Key words = erythromycin)) |
| PubMed | (Fetal Membranes, Premature Rupture[MeSH Terms] or Prelabor rupture of membranes[Title/Abstract]) AND (Anti-Infective Agents[MeSH Terms] or Antibiotics[Title/Abstract] OR Penicillins[MeSH Terms] OR Penicillins[Title/Abstract] OR Cephalosporins[MeSH Terms] OR Cephalosporins[Title/Abstract] OR azithromycin[MeSH Terms] OR azithromycin[Title/Abstract] OR erythromycin[MeSH Terms] OR erythromycin[Title/Abstract] OR Clindamycin[MeSH Terms] OR Clindamycin [Title/Abstract]) |
| Embase（via OvidSP） | 1 exp premature fetus membrane rupture/  2 premature fetus membrane rupture.ab.  3 exp antiinfective agent/  4 exp penicillin derivative/  5 exp cephalosporin derivative/  6 exp azithromycin/  7 exp erythromycin/  8 exp clindamycin/  9 (antibiotics or penicillins or cephalosporins or azithromycin or erythromycin or clindamycin).ab.  10 1 or 2  11 3 or 4 or 5 or 6 or 7 or 8 or 9  12 10 and 11 |
| Cochrane Library | #1 MeSH descriptor: [Fetal Membranes, Premature Rupture] explode all trees  #2 (Prelabor rupture of membranes): ti,ab,kw  #3 MeSH descriptor: [Anti-Infective Agents] explode all trees  #4 MeSH descriptor: [Penicillins] explode all trees  #5 MeSH descriptor: [Cephalosporins] explode all trees  #6 MeSH descriptor: [Azithromycin] explode all trees  #7 MeSH descriptor: [Erythromycin] explode all trees  #8 MeSH descriptor: [Clindamycin] explode all trees  #9 (“antibiotics” or “penicillins” or “cephalosporins” or “azithromycin” or “erythromycin” or “clindamycin”): ti,ab,kw  #10 #1 or #2  #11 #3 OR #4 OR #5 OR #6 OR #7 OR #8 OR #9  #12 #10 AND #11 |

**S2 table: characteristics of included studies**

| **Study ID** | **Location** | **Study design** | **PROM time for participants** | **Interventions** | **Composite outcome** | **Number of outcomes** | **Definition** | **Measurement** | **Reference ID** |
| --- | --- | --- | --- | --- | --- | --- | --- | --- | --- |
| A 2021 | China | cohort study | TPROM | different timing of antibiotics administration |  | 7 | √ |  | 22 |
| Ali 2020 | Egypt | cohort study | PPROM | different antibiotics |  | 4 |  |  | 23 |
| Almeida 1996 | Mozambique | RCT | PPROM | using antibiotics vs. placebo/blank control |  | 5 |  |  | 24 |
| Amon 1988 | the United States | RCT | PPROM | using antibiotics vs. placebo/blank control |  | 3 | √ |  | 25 |
| Bar 2000 | the United States | cohort study | PPROM | using antibiotics vs. placebo/blank control |  | 11 | √ |  | 26 |
| Barisic 2017 | Yugoslavia | cohort study | TPROM | different timing of antibiotics administration |  | 6 |  |  | 27 |
| Bergström 1991 | Mozambique | cohort study | PPROM | using antibiotics vs. placebo/blank control |  | 4 |  |  | 28 |
| Brelje 1966 | the United States | RCT | NR | using antibiotics vs. placebo/blank control |  | 7 | √ |  | 29 |
| Chang 2017 | Korea | cohort study | PPROM | using antibiotics vs. placebo/blank control | √ | 17 | √ | √ | 30 |
| Chatzakis 2020 | Greece | systematic review | PPROM | different antibiotics |  | 9 | √ |  | 31 |
| Chen 2020 | China | cohort study | PPROM | different timing of antibiotics administration |  | 5 |  |  | 32 |
| Chen 2021 | China | RCT | PPROM | antibiotics chosen depending on experience vs. culture results |  | 6 |  |  | 33 |
| Cong 2021 | China | RCT | PPROM | antibiotics chosen depending on experience vs. culture results |  | 3 |  |  | 34 |
| Cousens 2010 | the United Kingdom | systematic review | PPROM | using antibiotics vs. placebo/blank control |  | 5 |  |  | 35 |
| David 1995 | the United States | RCT | PPROM | different antibiotics |  | 19 | √ |  | 36 |
| Deng 2020 | China | RCT | PPROM | different timing of antibiotics administration |  | 3 |  |  | 37 |
| Dotters-Katz 2017 | the United States | cohort study | PPROM | using antibiotics vs. placebo/blank control |  | 4 | √ |  | 38 |
| Du 2016 | China | cohort study | PPROM | using antibiotics vs. placebo/blank control |  | 5 |  |  | 39 |
| Du 2019 | China | cohort study | PPROM | different timing of antibiotics administration |  | 7 |  |  | 40 |
| Du 2020 | China | cohort study | PPROM | different timing of antibiotics administration |  | 7 |  |  | 41 |
| Ehsanipoor 2008 | the United States | cohort study | PPROM | different antibiotics |  | 6 | √ |  | 42 |
| Ernest 1994 | the United States | RCT | PPROM | using antibiotics vs. placebo/blank control |  | 6 | √ |  | 43 |
| Feng 2020 | China | cohort study | PPROM | using antibiotics vs. placebo/blank control |  | 12 |  |  | 44 |
| Finneran 2019 | the United States | cohort study | PPROM | different antibiotics |  | 1 |  |  | 45 |
| Finneran 2017 | the United States | cohort study | PPROM | different antibiotics |  | 12 |  |  | 46 |
| Fitzgibbon 2021 | Israel | cohort study | PPROM | different antibiotics |  | 5 | √ |  | 47 |
| Fuhr 2006 | Germany | RCT | PPROM | using antibiotics vs. placebo/blank control |  | 5 |  |  | 48 |
| Grable 1996 | the United States | RCT | PPROM | using antibiotics vs. placebo/blank control |  | 11 | √ |  | 49 |
| Heine 2019 | the United States | RCT | PPROM | different antibiotics | √ | 4 | √ |  | 50 |
| Johnston 1990 | the United States | RCT | PPROM | using antibiotics vs. placebo/blank control |  | 21 |  |  | 51 |
| Kahramanoglu 2016 | Turkey | RCT | PPROM | different antibiotics | √ | 17 | √ |  | 52 |
| Ke 2013 | China | cohort study | PPROM | using antibiotics vs. placebo/blank control |  | 5 |  |  | 53 |
| Kenyon 2001 | the United Kingdom | RCT | PPROM | different antibiotics | √ | 15 | √ |  | 54 |
| Kenyon 2004 | the United Kingdom | systematic review | PPROM | using antibiotics vs. placebo/blank control |  | 6 |  |  | 55 |
| Kenyon 2008 | the United Kingdom | cohort study | NR | different antibiotics | √ | 10 | √ | √ | 56 |
| Knupp 2022 | the United States | cohort study | PPROM | different timing of antibiotics administration | √ | 7 | √ |  | 57 |
| Kole-White 2021 | the United States | cohort study | PPROM | different administration route |  | 3 | √ |  | 58 |
| Kurki 1992 | Finland | RCT | PPROM | using antibiotics vs. placebo/blank control |  | 13 | √ |  | 59 |
| Kwak 2013 | Korea | RCT | both TPROM and PPROM | different antibiotics | √ | 3 | √ | √ | 60 |
| Lee 2016 | Korea | cohort study | PPROM | different antibiotics |  | 16 |  |  | 61 |
| Li 2015 | China | cohort study | PPROM | different timing of antibiotics administration |  | 4 |  |  | 62 |
| Li 2020 | China | cohort study | PPROM | different courses of antibiotics administration |  | 4 |  |  | 63 |
| Li 2021 | China | cohort study | PPROM | antibiotics chosen depending on experience vs. culture results |  | 3 |  |  | 64 |
| Liang 2018 | China | RCT | PPROM | different timing of antibiotics administration |  | 8 |  |  | 65 |
| Lin 2012 | China | cohort study | PPROM | using antibiotics vs. placebo/blank control |  | 6 |  |  | 66 |
| Lockwood 1993 | the United States | RCT | PPROM | using antibiotics vs. placebo/blank control | √ | 12 | √ |  | 67 |
| Lovett 1997 | the United States | RCT | PPROM | different antibiotics |  | 13 |  |  | 68 |
| M. Siegel 2019 | the United States | cohort study | PPROM | different antibiotics |  | 7 | √ |  | 69 |
| Mai 2016 | China | RCT | PPROM | antibiotics chosen depending on experience vs. culture results |  | 2 |  |  | 70 |
| Martingano 2020 | the United States | cohort study | PPROM | different antibiotics |  | 6 | √ |  | 71 |
| Maymon 1998 | Israel | systematic review | PPROM | using antibiotics vs. placebo/blank control |  | 8 |  |  | 72 |
| McCaul 1992 | the United States | RCT | PPROM | using antibiotics vs. placebo/blank control |  | 7 |  |  | 73 |
| McGregor 1991 | the United States | RCT | PPROM | different antibiotics |  | 13 |  |  | 74 |
| Mercer 1992 | the United States | RCT | PPROM | using antibiotics vs. placebo/blank control |  | 19 | √ |  | 75 |
| Mercer 1995 | the United States | systematic review | PPROM | using antibiotics vs. placebo/blank control |  | 10 |  |  | 76 |
| Moore 2003 | the United States | RCT | PPROM | different courses of antibiotics administration |  | 9 |  |  | 77 |
| Nabhan 2014 | Egypt | RCT | both TPROM and PPROM | using antibiotics vs. placebo/blank control |  | 13 | √ |  | 78 |
| Navathe 2019 | the United States | cohort study | PPROM | different antibiotics |  | 12 |  |  | 79 |
| Ovalle 2002 | Chile | RCT | PPROM | using antibiotics vs. placebo/blank control |  | 3 |  |  | 80 |
| Pan 2018 | China | cohort study | PPROM | different timing of antibiotics administration |  | 9 |  |  | 81 |
| Pasquier 2019 | Canada | RCT | PPROM | using antibiotics vs. placebo/blank control | √ | 2 | √ |  | 82 |
| Pawar 2020 | India | cohort study | PPROM | different antibiotics |  | 10 |  |  | 83 |
| Pierson 2014 | the United States | cohort study | PPROM | different antibiotics |  | 8 | √ |  | 84 |
| Rodney 2000 | the United States | cohort study | PPROM | different antibiotics |  | 3 |  |  | 85 |
| Ryo 2005 | Japan | cohort study | PPROM | different antibiotics |  | 3 |  |  | 86 |
| Saccone 2015 | the United States | systematic review | both TPROM and PPROM | using antibiotics vs. placebo/blank control |  | 21 | √ |  | 87 |
| Segel 2003 | the United States | RCT | PPROM | different courses of antibiotics administration | √ | 5 | √ |  | 88 |
| Smith 2015 | the United States | cohort study | PPROM | different courses of antibiotics administration | √ | 3 | √ |  | 89 |
| Song 2005 | Korea | cohort study | PPROM | using antibiotics vs. placebo/blank control |  | 6 |  |  | 90 |
| Sung 2017 | the United States | cohort study | PPROM | different antibiotics |  | 8 | √ |  | 91 |
| Tai 2011 | China | cohort study | TPROM | different timing of antibiotics administration |  | 5 |  |  | 92 |
| Tsumura 2019 | Japan | cohort study | PPROM | different antibiotics |  | 24 |  |  | 93 |
| Wayne 1996 | the United States | cohort study | PPROM | using antibiotics vs. placebo/blank control |  | 5 | √ |  | 94 |
| Wojcieszek 2014 | the United Kingdom | systematic review | both TPROM and PPROM | using antibiotics vs. placebo/blank control | √ | 31 |  |  | 95 |
| Wolf 2020 | Israel | RCT | PPROM | different antibiotics |  | 7 |  |  | 96 |
| Wu 2018 | China | cohort study | PPROM | different antibiotics |  | 7 |  |  | 97 |
| Yeung 2014 | China | cohort study | PPROM | different antibiotics |  | 3 |  |  | 98 |
| Zeng 2020 | China | cohort study | PPROM | different timing of antibiotics administration |  | 9 |  |  | 99 |
| Zhang 2014 | China | RCT | PPROM | using antibiotics vs. placebo/blank control |  | 7 |  |  | 100 |
| Zhang 2017 | China | cohort study | PPROM | antibiotics chosen depending on experience vs. culture results |  | 7 |  |  | 101 |
| Zhang 2019 | China | cohort study | PPROM | different timing of antibiotics administration |  | 4 |  |  | 102 |
| Zhao LJ 2019 | China | cohort study | PPROM | different antibiotics |  | 9 |  |  | 103 |
| Zhao WJ 2019 | China | cohort study | TPROM | different timing of antibiotics administration | √ | 6 | √ |  | 104 |
| Zheng 2016 | China | cohort study | PPROM | different antibiotics | √ | 12 | √ |  | 105 |
| Zheng 2018 | China | RCT | TPROM | different timing of antibiotics administration |  | 5 |  |  | 106 |
| Zheng 2020 | China | cohort study | TPROM | different timing of antibiotics administration | √ | 10 | √ |  | 107 |
| Zheng 2021 | China | RCT | PPROM | antibiotics chosen depending on experience vs. culture results |  | 5 |  |  | 108 |
| Zhou 2015 | China | cohort study | PPROM | different timing of antibiotics administration |  | 4 |  |  | 109 |
| Zhou 2020 | China | cohort study | PPROM | antibiotics chosen depending on experience vs. culture results |  | 7 |  |  | 110 |
| Zou 2021 | China | cohort study | PPROM | antibiotics chosen depending on experience vs. culture results |  | 6 |  |  | 111 |

NR: not reported

**S3 table: socioeconomic information of participants**

| ID | Age | Gestational weeks（PROM） | Gravidity and parity history | Occupation |
| --- | --- | --- | --- | --- |
| 1 | 25 | 31+5 | G1P0 | others |
| 2 | 35 | 37 | G1P0 | freelancer |
| 3 | 25 | 28+5 | G1P0 | others |
| 4 | 36 | 38 | G1P0 | bank staff |
| 5 | 33 | 36+1 | G1P0 | company employee |
| 6 | 34 | 32+1 | G5P1+3 | others |
| 7 | 25 | 29+1 | G7P0+6 | famer |
| 8 | 27 | 15+4 | G1P0 | professionals |
| 9 | 44 | 37+2 | G2P0+1 | bank staff |
| 10 | 34 | 39+6 | G3P0+2 | others |
| 11 | 35 | 38 | G1P0 | others |
| 12 | 33 | 37+1 | G1P0 | others |
| 13 | 29 | 39+1 | G1P0 | company employee |
| 14 | 32 | 28+4 | G1P0 | others |
| 15 | 30 | 34+1 | G2P0+1 | company employee |
| 16 | 28 | 35+4 | G1P0 | others |
| 17 | 29 | 39+3 | G1P0 | company employee |
| 18 | 23 | 26+1 | G5P0+4 | others |
| 19 | 31 | 25+3 | G1P0 | professionals |
| 20 | 31 | 32+4 | G2P0+1 | others |
| 21 | 35 | 19+1 | G9P0+8 | freelancer |
| 22 | 28 | 33+3 | G2P1 | others |
| 23 | 34 | 20 | G2P1 | others |
| 24 | 30 | 28+5 | G1P0 | self-employed |
| 25 | 29 | 36+6 | G1P0 | company employee |
| 26 | 31 | 39+6 | G3P0+2 | others |
| 27 | 36 | 32 | G1P0 | national public servant |
| 28 | 28 | 40+2 | G1P0 | others |
| 29 | 31 | 28+4 | G1P0 | others |
| 30 | 29 | 34+1 | G3P0+2 | others |
